# DDIT3/CHOP and the sarcoma fusion oncoprotein FUS-DDIT3/TLS-CHOP bind cyclin-dependent kinase 2

**DOI:** 10.1186/1471-2121-10-89

**Published:** 2009-12-17

**Authors:** Christoffer Bento, Mattias K Andersson, Pierre Åman

**Affiliations:** 1 Lundberg Laboratory for Cancer Research, Department of Pathology, University of Gothenburg, Gothenburg, Sweden

## Abstract

**Background:**

The *DDIT3 *gene encodes a transcription factor belonging to the CCAAT/enhancer binding protein (C/EBP) family. It is normally expressed at very low levels but is activated by cellular stress conditions and induces G1 arrest and, in some cell types, apoptosis. *DDIT3 *is found as a part of the fusion oncogene *FUS-DDIT3 *that is causal for the development of myxoid/round-cell liposarcomas (MLS/RCLS).

**Results:**

In the present study, we searched for putative interaction partners of DDIT3 and the oncogenic FUS-DDIT3 among G1 cyclins and cyclin-dependent kinases. We found that FUS-DDIT3 and the normal DDIT3 bind CDK2. In addition, CDK2 showed an increased affinity for cytoskeletal proteins in cells expressing FUS-DDIT3 and DDIT3.

**Conclusions:**

We conclude that DDIT3 binds CDK2 and that many of the observed biological effects of DDIT3 may involve interaction with CDK2.

## Background

*DDIT3 *(*GADD153, CHOP*) encodes a transcription factor belonging to the CCAAT/enhancer binding protein (C/EBP) family [1]. *DDIT3 *is normally transcribed at very low levels but is elevated upon DNA damage in cellular stress conditions [2-6]. The DDIT3 protein has a central role in endoplasmatic reticulum stress and DNA damage response by inducing cell cycle arrest and apoptosis [7,8]. DDIT3 has recently been implicated in the stress response leading to death of pancreatic insulin producing β-cells [9] and it may also be a part of cellular stress conditions causing neurodegenerative disorders [10]. In addition, DDIT3 is believed to be involved in growth cessation and terminal differentiation of lipoblasts, osteoblasts and erythrocytes [11-13]. DDIT3 forms heterodimers with several other C/EBP family members [14] as well as other leucine zipper carrying proteins [15,16] and the heterodimers are believed to act as dominant negative inhibitors of transcription [14].

*DDIT3 *is also a part of a fusion oncogene critical for the development of myxoid/round cell liposarcoma (MLS/RCLS) [17,18]. The tumor cells carry the chromosomal translocation t(12;16)(q13;p11) that results in fusion of *DDIT3 *to *FUS *(also called *TLS*) or more rarely the t(12;22)(q13;q12) that fuses *DDIT3 *to *EWSR1 *[19-21]. The chimerical FUS-DDIT3 oncoprotein functions as an abnormal transcription factor [22] and localizes to distinct nuclear structures in cultured cells [23,24]. MLS/RCLS cells exhibit abnormal expression profiles of cell cycle controlling factors and among them cyclins and cyclin-dependent kinases (CDKs) [25]. In addition, the DDIT3-binding C/EBPα has been shown to interact and inhibit the kinase activity of CDK2 and CDK4 [26]. Based on these observations, we searched for putative interaction partners of the oncogenic FUS-DDIT3 with G1 cyclins and CDKs as these might provide insight into the molecular mechanisms by which the chimerical oncoprotein induces malignancy.

## Results

### CDK2 and cyclin E colocalize with FUS-DDIT3

**Figure 1:**
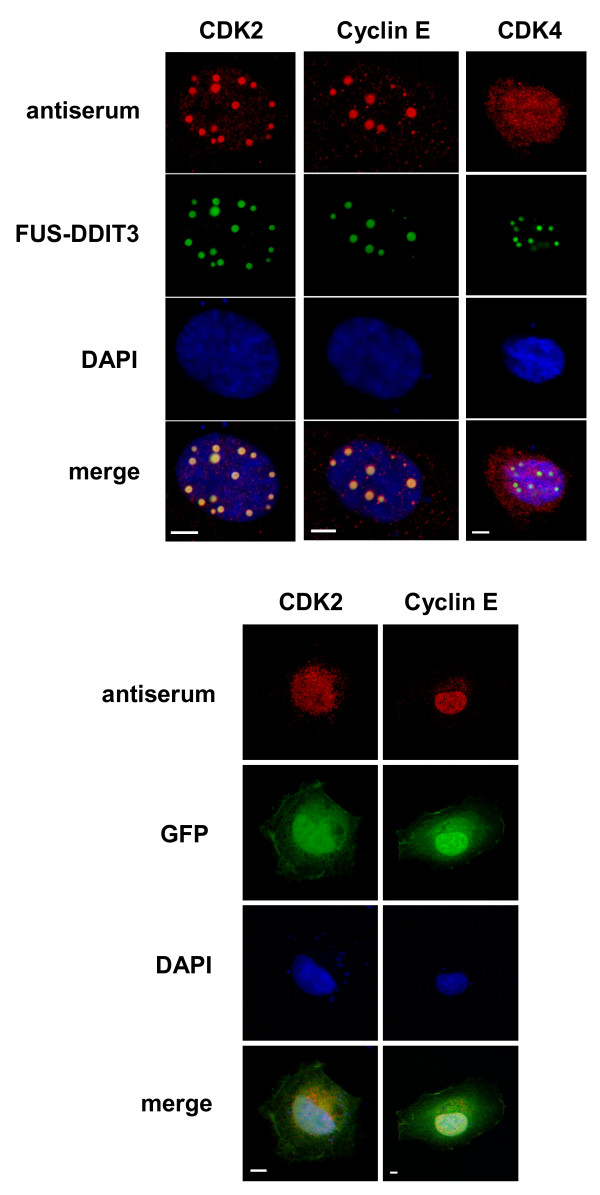
**CDK2 and cyclin E colocalize with FUS-DDIT3 in transiently transfected HT1080 cells**. The endogenous distribution of CDK2 and cyclin E seen in red is detected by antiserum specific for these proteins. Ectopically expressed GFP-tagged FUS-DDIT3 is shown in green. The DAPI dye is used to stain nuclei blue. CDK4 was not seen to accumulate in FUS-DDIT3-containing granules and no granules were formed in cells overexpressing the GFP protein only. Bars indicate 5 μm.

HT1080 cells were transiently transfected with a *FUS-DDIT3*-GFP construct and colocalization between the ectopically expressed fusion oncoprotein and endogenous cyclin D1, cyclin E, CDK2 and CDK4 was investigated by immunofluorescence. Cyclin E and CDK2 showed prominent colocalization with the FUS-DDIT3 protein and were detected in FUS-DDIT3 containing granules in the majority of cells (Figure 1). We found no signs of colocalization between FUS-DDIT3 and CDK4 (Figure 1) or cyclin D1 (not shown). Cells transfected with the empty GFP vector showed no granules and a smooth nuclear distribution of CDK2 and cyclin E (Figure 1).

### FUS-DDIT3 binds CDK2 through its DDIT3 part

**Figure 2:**
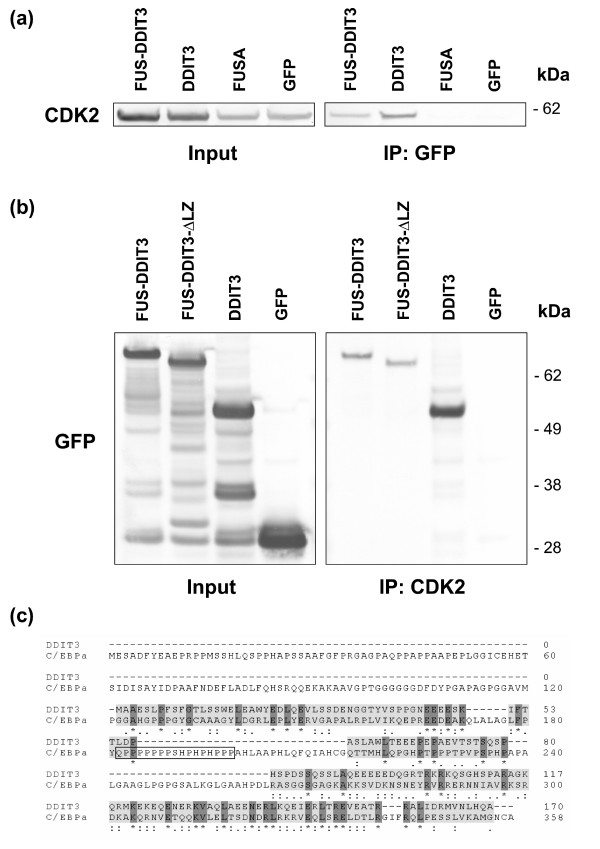
**Co-immunoprecipitation of DDIT3 and CDK2**. **(a) **HT1080 cells were co-transfected with the indicated GFP constructs and CDK2-DsRed1. Proteins in cell lysates were further immunoprecipitated with anti GFP serum and recombinant CDK2-DsRed1 protein was detected by western blot using CDK2 antibodies. Input shows proteins before immunoprecipitation of cell lysates while IP shows proteins present in immunoprecipitates. **(b) **HT1080 cells were transiently transfected with the indicated GFP constructs and cellular proteins were immunoprecipitated with CDK2 antibodies. Recombinant proteins in samples were detected with anti GFP serum. The most intense bands in the input show the correct size of the GFP-tagged protein products while additional unspecific bands were also reacting with the GFP antisera in these samples. **(c) **ClustalW alignment of DDIT3 and C/EBPα amino acid sequences. A region shown to bind CDK2 in C/EBPα is boxed. Letters indicate standard amino acid abbreviations. Amino acids with related physicochemical characteristics are displayed below the sequences as: '*' (identical residue), ':' (highly similar residue), '.' (weakly similar residue). The leucine zipper region is seen towards the C-terminal in both proteins.

Cells were transfected with *FUS-DDIT3*-GFP and cellular proteins were immunoprecipitated with GFP antibodies and further analyzed by western blot. In pilot experiments, we used HT1080 cells stably expressing FUS-DDIT3-GFP and could weakly detect endogenous CDK2 in immunoprecipitates while cyclin E was not found (data not shown). To further confirm a possible interaction between FUS-DDIT3 and CDK2, we instead transiently transfected cells using cloned *CDK2 *cDNA expressed in frame with the fluorescent protein DsRed1. Cells co-transfected with *CDK2*-DsRed1 and either of *FUS-DDIT3 *or *DDIT3 *expressing constructs showed presence of CDK2-DsRed1 in anti-GFP immunoprecipitates from both FUS-DDIT3 and DDIT3 transfected cells (Figure 2a). In contrast, CDK2-DsRed1 was not found in immunoprecipitates of cells transfected with the N-terminal part of FUS present in the FUS-DDIT3 protein or in GFP-transfected control cells (Figure 2a). In a reverse experiment, HT1080 cells were transfected with *FUS-DDIT3*, *FUS-DDIT3ΔLZ*, *DDIT3 *and GFP constructs and cellular proteins in extracts were immunoprecipitated with anti CDK2 antibodies (Figure 2b). We found that endogenous CDK2 co-immunoprecipitated with DDIT3, FUS-DDIT3 and FUS-DDIT3 lacking a leucine zipper domain. These results indicate that the region binding CDK2 is located N-terminally of the leucine zipper part of DDIT3. ClustalW alignment performed between DDIT3 and the related C/EBPα showed conservation of several amino acids in distinct regions shared by the two proteins but that DDIT3 lacks a region similar to the one that binds CDK2 in C/EBPα [26] (Figure 2c).

### CDK2 expression, phosphorylation status and turnover is not changed by FUS-DDIT3

**Figure 3:**
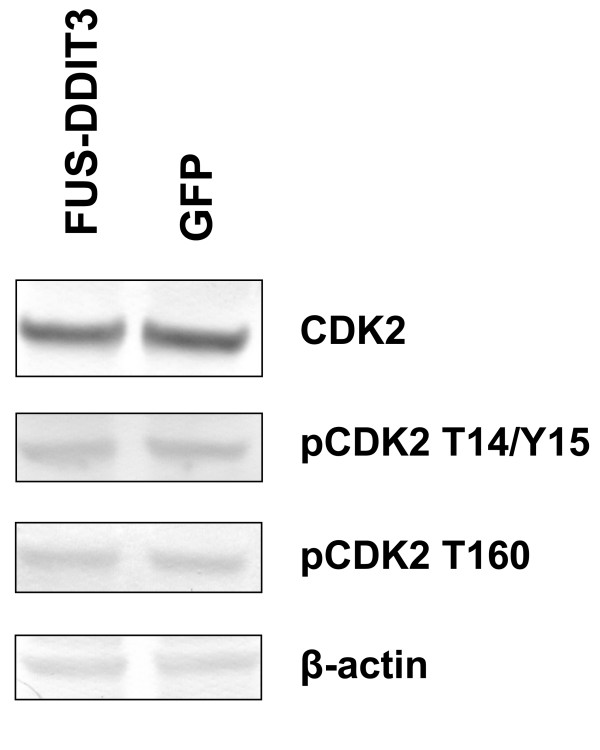
**Expression and phosphorylation status of CDK2**. Western blot analysis of cell lysates from HT1080 cells transiently transfected with *FUS-DDIT3*-GFP and GFP. β-actin is used as loading control.

The CDK2 expression in HT1080 cells after 42 hours of transfection with *FUS-DDIT3*-GFP or GFP was investigated by western blot analysis (Figure 3). In addition, the phosphorylation status of CDK2 was analyzed using CDK2 antibodies targeting either the two inhibitory phosphorylations at threonine 14 and tyrosine 15 or the activating phosphorylation at threonine 160 (Figure 3). No apparent difference in CDK2 phosphorylation between cells expressing FUS-DDIT3-GFP or GFP was detected and the amount of CDK2 between the two transfected cell populations was equivalent (Figure 3). The CDK2 protein half-life did not differ between stably transfected FUS-DDIT3 cells and HT1080 control cells upon a six hour cycloheximide chase assay (not shown).

### CDK2 protein binding affinity is altered in FUS-DDIT3 and DDIT3 expressing cells

**Figure 4:**
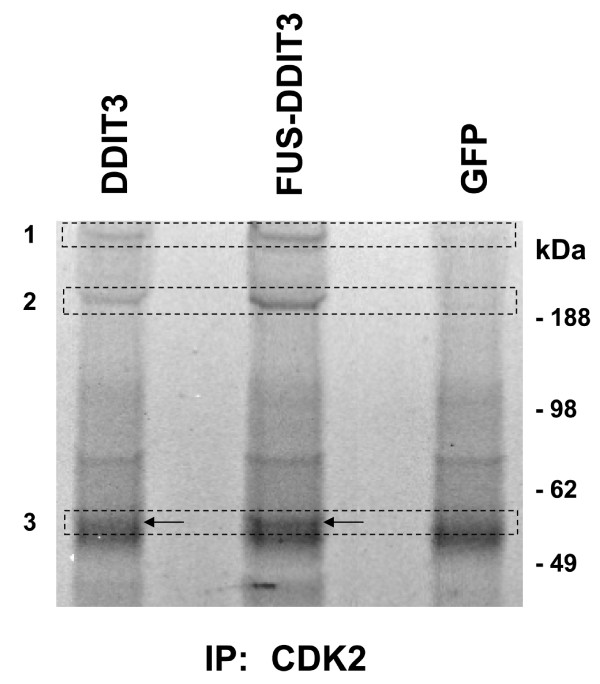
**SYPRO stained gel of co-immunoprecipitated proteins in extracts from HT1080 cells transiently transfected with either of *DDIT3*-GFP, *FUS-DDIT3*-GFP or GFP constructs**. CDK2 antibodies were used for immunoprecipitations. Boxes indicate regions of the gel analyzed by mass spectrometry. Three proteins were enriched in immunoprecipitates from DDIT3 and FUS-DDIT3 expressing cells (region 1: plectin, region 2: myosin, region 3: vimentin). Arrows indicate the differentially immunoprecipitated vimentin bands present in lanes of DDIT3 and FUS-DDIT3 cells but absent in that of GFP cells. These bands migrate slightly slower than the thicker bands inside the same region. The thicker bands were identified as rabbit heavy chain immunoglobulin molecules and these originate from the antibodies used for immunoprecipitation of CDK2 and are present in all three lanes.

HT1080 cells were transfected with either of *FUS-DDIT3*, *DDIT3 *or GFP and CDK2 was immunoprecipitated from lysates with CDK2 specific antibodies (Figure 4). Precipitates were analyzed by SDS polyacrylamide gel electrophoresis and stained with Coomassie Blue. Several bands were present in the FUS-DDIT3 and DDIT3 precipitates that were absent in precipitates from GFP expressing cells (Figure 4). Bands from three regions were cut as indicated in the figure and analyzed by LC-MS/MS. The cytoskeletal cross-linker plectin (Figure 4, region 1), the actin motor-protein myosin (Figure 4, region 2) and the intermediary filament protein vimentin (Figure 4, region 3) were enriched in immunoprecipitates of FUS-DDIT3 and DDIT3 cells. The thicker bands with similar abundance in all three lanes in region 3 were identified by mass spectrometry as rabbit heavy chain immunoglobulin molecules.

## Discussion

In the present study, we used immunofluorescence microscopy to investigate putative colocalization between FUS-DDIT3 and cyclins/cyclin-dependent kinases involved in G1 cell cycle control. CDK2 and the CDK2 binding cyclin E were found to change their localization in FUS-DDIT3 expressing cells to a pattern that was identical to the FUS-DDIT3 nuclear granules reported earlier [24]. This suggests that CDK2 and cyclin E are translocated to nuclear structures defined by the FUS-DDIT3 protein. As the DDIT3 part is considered a DNA-binding transcription factor [27], we speculate that the FUS-DDIT3 defined structures may contain active chromatin and mRNA at different stages of processing. Thus, the accumulation of CDK2 and cyclin E to such nuclear regions may result in changed phosphorylation patterns and regulation of substrates present at these foci.

DDIT3 is able to form DNA-binding heterodimers with C/EBPα through its leucine zipper region [14] and C/EBPα is reported to bind CDK2 and inhibit its kinase activity [26]. Therefore, it is possible that the binding between DDIT3 and CDK2 is mediated through C/EBPα or another C/EBP protein. However, we here show that a DDIT3 mutant lacking the leucine zipper domain binds CDK2, which implies a binding between DDIT3 and CDK2 that is independent of other C/EBP proteins. DDIT3 and C/EBPα contain several regions with sequence similarities but the proline-histidine rich part reported to bind CDK2 in C/EBPα [26] (Figure 2c) is not present in DDIT3. This suggests that DDIT3 binds CDK2 through a different mechanism than C/EBPα.

Analyses of GFP-immunoprecipitates failed to detect cyclin E in these samples. The absence of cyclin E in CDK2 immunoprecipitates bears resemblance to previous reports showing that C/EBPα disrupts CDK2/cyclin complexes leading to growth arrest [26]. Hence, it is possible that the DDIT3 bound to CDK2 disrupts CDK2/cyclin E complexes in similar way as C/EBPα. Consequently, since cyclin E is a major regulator of CDK2 kinase activity in G1, the DDIT3 binding may alter CDK2 activity in DDIT3 and FUS-DDIT3 expressing cells. We did however not detect a change in phosphorylation status of CDK2 in FUS-DDIT3 expressing cells compared to control cells. To further analyze the functional effects of DDIT3/FUS-DDIT3 binding to CDK2, we immunoprecipitated CDK2 in cells transiently transfected with *FUS-DDIT3*, *DDIT3 *and GFP constructs. Analysis of the precipitates revealed enhanced binding of CDK2 to components of the cytoskeleton in cells expressing FUS-DDIT3 and DDIT3. An increased affinity for cytoskeletal components and crosstalk between cell cycle proteins and cytoskeletal regulatory proteins could lead to changes in cytoskeleton structure [28].

## Conclusions

In conclusion, we show that CDK2 is translocated to nuclear structures defined by the FUS-DDIT3 oncoprotein and that it binds the DDIT3 part of the chimera. Cyclin E is also recruited to FUS-DDIT3 nuclear structures but can not be found in CDK2-containing DDIT3/FUS-DDIT3 precipitates. The interaction of FUS-DDIT3 and DDIT3 with CDK2 appears to alter the binding affinity of CDK2, possibly leading to changed phosphorylation patterns and regulation of cytoskeletal or other proteins. Many of the observed biological effects of DDIT3 may involve the interaction with CDK2.

## Methods

### Cell culture and transfection

Human HT1080 fibrosarcoma cells were grown in RPMI 1640 medium (Sigma-Aldrich) supplied with 10% fetal bovine serum, penicillin (50 U/ml) and streptomycin (50 μg/ml) at 37°C in 5% CO_2_. Cells were transiently transfected at 50-70% confluence using the FuGENE 6 transfection reagent (Roche) with a 3:1 FuGENE:DNA ratio, according to the instructions supplied by the manufacturer.

### Immunofluorescence

Cells were fixed 24 hours after transfection in 3.7% formaldehyde in PBS for 10 min, rinsed in PBS and stained with rabbit polyclonal antibodies for CDK2 (C5223, Sigma-Aldrich), CDK4 (C8218, Sigma-Aldrich), cyclin D1 (M7155, Dako) or cyclin E (C4976, Sigma-Aldrich) that were detected using Cy3 conjugated secondary antibodies (Fluorolink, Amersham Biosciences). Finally, slides were mounted using DAPI/DABCO solution (Sigma-Aldrich), incubated over night at room temperature and then imaged using a Zeiss LSM510 META confocal microscope system.

### Expression vectors

For cloning of CDK2 the following primers were used: CDK2 forward GATCTCGAGCCACCATGGAGAACTTCCAAAAG, CDK2 reverse CAAGGATCCCGGAGTCGAAGATGGGGTACT. The CDK2 fragment was cloned into the pDsRed1-N1 vector (Clontech Laboratories) in frame with the DsRed1 sequence. All constructs were sequenced to exclude mutant clones. *FUS-DDIT3*-GFP, *DDIT3*-GFP and *FUSA*-GFP were previously described [24,29]. The *FUS-DDIT3ΔLZ*-GFP construct, expressing a protein lacking the 38 most C-terminal amino acids (including the leucine zipper region), is described elsewhere [30]. Stably transfected FUS-DDIT3 cells has been described previously [31].

### Co-immunoprecipitation

HT1080 cells were co-transfected with CDK2-DsRed and/or FUS-DDIT3-GFP/FUS-DDIT3-ΔLZ/DDIT3-GFP/FUSA-GFP/GFP constructs at 50-70% confluence. Twenty-four hours post transfection, cells were washed in PBS, scraped and lysed in NP-40 buffer (150 mM NaCl, 1% NP-40, 50 mM Tris pH 8.0) containing protease inhibitor (Complete Mini, Roche). Debris was removed by centrifugation at 14,000 g and supernatants were used for co-immunoprecipitations. Two microgram of antibodies specific for GFP (8372-2, BD Pharmingen) or CDK2 (C5223, Sigma-Aldrich) were prebound to protein A agarose beads (Millipore) blocked with 5% bovine serum albumin in NP-40 buffer overnight. Cell lysates were incubated with antibody-beads mixtures for 4 h at room temperature. Beads were washed 5 times with NP-40 buffer and proteins were released from beads by addition of 4× NuPAGE LDS sample buffer (Invitrogen) and incubation at 95°C. Supernatants were subsequently analyzed with western blot or with SYPRO Ruby gel staining (Sigma-Aldrich) according to the instructions supplied by the manufacturer. SYPRO stained gels were analyzed by liquid chromatography-mass spectrometry (LC-MS/MS) at the Proteomics Core Facility at University of Gothenburg. For experiments with phospho-CDK2 antibodies (see below), transfected cells were lysed in RIPA buffer (50 mM Tris-HCl pH 7.4, 150 mM NaCl, 1 mM EDTA, 1% Triton x-100, 1% Sodium deoxycholate, 0.1% SDS) containing protease inhibitors and phosphatase inhibitors (NaF 10 mM, Na_3_VO_4 _1 mM).

### Western Blot Analysis

Proteins were separated using NuPAGE 4-12% Bis-Tris gels (Invitrogen), blotted onto PVDF membranes (Millipore) and probed with the following primary antibodies: CDK2 (sc-6248, Santa Cruz Biotechnology), p-CDK2 Thr14/Tyr15 (Santa Cruz Biotechnology), p-CDK2 Thr160 (Cell Signaling), cyclin E (C4976, Sigma-Aldrich), β-actin (mAbcam8226, Abcam) and GFP (8371-2, BD Pharmingen). Primary antibodies were detected with alkaline phosphatase conjugated secondary antibodies (Dako) and bands were visualized with BCIP/NBT Alkaline Phosphatase Substrate (Sigma-Aldrich).

### ClustalW alignment

The amino acid sequences of DDIT3 and C/EBPα were aligned using the ClustalW algorithm available at the Universal protein resource (UniProt) [32].

## Abbreviations

CDK2: cyclin-dependent kinase 2; C/EBP: CCAAT/enhancer binding protein; CHOP: C/EBP homologous protein; DDIT3: DNA damage-inducible transcript 3; FUS: fusion; GFP: green fluorescent protein; LC-MS/MS: liquid chromatography mass spectrometry; MLS/RCLS: myxoid/round-cell liposarcoma; TLS: translocated in liposarcoma.

## Authors' contributions

PÅ conceived of the study. CB, MKA and PÅ designed the experiments. CB and MKA performed research. CB, MKA and PÅ wrote the manuscript. All authors read and approved the final version of the manuscript.
